# Global-Scale Relationships between Colonization Ability and Range Size in Marine and Freshwater Fish

**DOI:** 10.1371/journal.pone.0049465

**Published:** 2012-11-21

**Authors:** Giovanni Strona, Paolo Galli, Simone Montano, Davide Seveso, Simone Fattorini

**Affiliations:** 1 Department of Biotechnology and Biosciences, University of Milano Bicocca, Milano, Italy; 2 Azorean Biodiversity Group and Platform for Enhancing Ecological Research & Sustainability (PEERS), Departamento de Ciências Agrárias Universidade dos Açores, Angra do Heroísmo, Portugal; The Australian National University, Australia

## Abstract

Although fish range sizes are expected to be associated with species dispersal ability, several studies failed to find a clear relationship between range size and duration of larval stage as a measure of dispersal potential. We investigated how six characteristics of the adult phase of fishes (maximum body length, growth rate, age at first maturity, life span, trophic level and frequency of occurrence) possibly associated with colonization ability correlate with range size in both freshwater and marine species at global scale. We used more than 12 million point records to estimate range size of 1829 freshwater species and 10068 marine species. As measures of range size we used both area of occupancy and extent of occurrence. Relationships between range size and species traits were assessed using Canonical Correlation Analysis. We found that frequency of occurrence and maximum body length had a strong influence on range size measures, which is consistent with patterns previously found (at smaller scales) in several other taxa. Freshwater and marine fishes showed striking similarities, suggesting the existence of common mechanisms regulating fish biogeography in the marine and freshwater realms.

## Introduction

To enlarge its distributional range, a species must reach a new, previously unoccupied area, and then succeed in colonizing it. The first process (dispersal) is mainly related to the species ability to cross unfavorable areas [1], whereas, the second (colonization) is determined by environmental constraints and species ecological requirements [2], [3]. Both processes are fundamental in determining species range size, so that a species with a high dispersal ability, but with low adaptability to new environments, may have a restricted range. Dispersal potential of marine fishes can be confidently estimated through the length of the larval phase [4], [5], [6], whereas the identification of possible determinants of dispersal ability in freshwater fishes is less straightforward. Freshwater species distributions are subject to a variety of constraints (such as dendritic arrangement of riverine ecosystems, changes in drainage basin boundaries, human alterations of river courses, sea water barriers, etc.), as well as to biogeographic patterns (such as the geological history of the areas), which make it difficult to disentangle the role of dispersal from the effects of environmental (hydrographical) and human-induced processes potentially responsible for species range expansion [7].

Because fish larvae can be transported by sea currents for hundreds to thousands of kilometers [8], dispersal ability is considered much more important than colonization ability in determining fish range size in the marine realm. A positive relationship between dispersal potential and range size is expected [5], yet several studies failed in finding a clear relationship between duration of the larval stage and range size in marine fishes [9]. However, these studies investigated only reef fish communities, which are problematic for various reasons. First, reef fish communities may be significantly sustained by endogenous recruitment [10]. Second, it is possible that the lack of habitat isolation in reefs obscures the relationship between dispersal and range size [9]. Third, it is difficult to establish if results obtained for reef fishes can be comparable to those obtained for species with different ecology, such as migratory (oceanodromous, anadromous, catadromous, potamodromous, amphidromous), demersal, and pelagic species.

On the other hand, range size expansion in freshwater fish species is generally attributed to geological changes to drainage basin boundaries, such as river captures and massive floods, and is therefore considered unrelated to species dispersal ability [7]. However, many freshwater species strictly associated with lentic waters could have virtually no connection with rivers. Moreover, river flow may afford excellent opportunity for long distance downstream dispersal of both larvae (during high discharge and directed water movement) and adults (as bi-directional migration routes).These difficulties in establishing robust theoretical linkages between fish dispersal ability and range size would lead to a serious reconsideration of the potential role of fish colonization ability in determining species distributions. Recent studies demonstrated that adult biology traits (especially environmental tolerance and body size) are important determinants of successful movements of reef fish across marine barriers [11] thus encouraging the possibility of relating range size to species traits at a global scale. The present paper makes use of the great amount of data on fish distribution and ecology provided respectively by Fishbase [12] and the Ocean Biogeographic System [13] to investigate how species traits possibly associated with colonization ability affect range size using a global scale approach and including also the freshwater realm.

**Figure 1:**
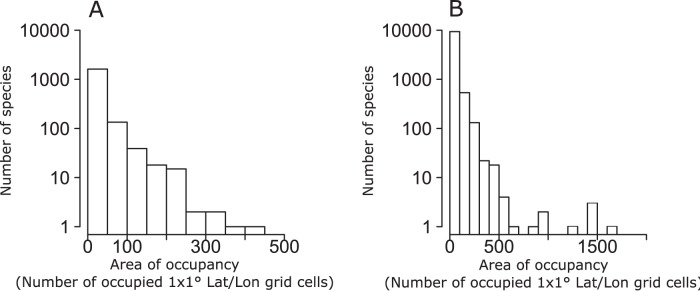
Frequency distribution plots of geographic range sizes expressed as area of occupancy (AOO, Number of occupied 1×1° Lat/Lon grid cells) in freshwater (A) and marine (B) fishes.

**Figure 2:**
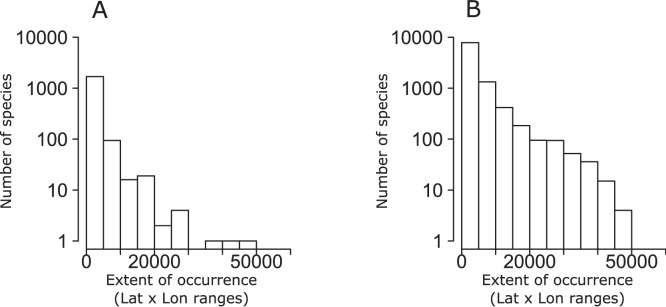
Frequency distribution plots of geographic range sizes expressed as extent of occurrence (EOO, number of grid cells of 1° along the maximum latitudinal range × number of grid cells of 1° along the maximum longitudinal range of each species) in freshwater (A) and marine (B) fishes.

## Materials and Methods

More than 12 million latitude/longitude point records for more than 11,000 bony fish species were retrieved from The Ocean Biogeographic Information System (OBIS) [13]. Biogeographic accuracy of OBIS data has been previously questioned [14], [15]. However, OBIS database is continuously updated with a constant increase in the number of its records, which has grown from around 15 million records in 2008 (the year it was heavily criticized [14], [15]), to almost 33 million records in 2012 [13]. This improvement has recently facilitated its use in several macroecological studies [16]–[19].

**Figure 3:**
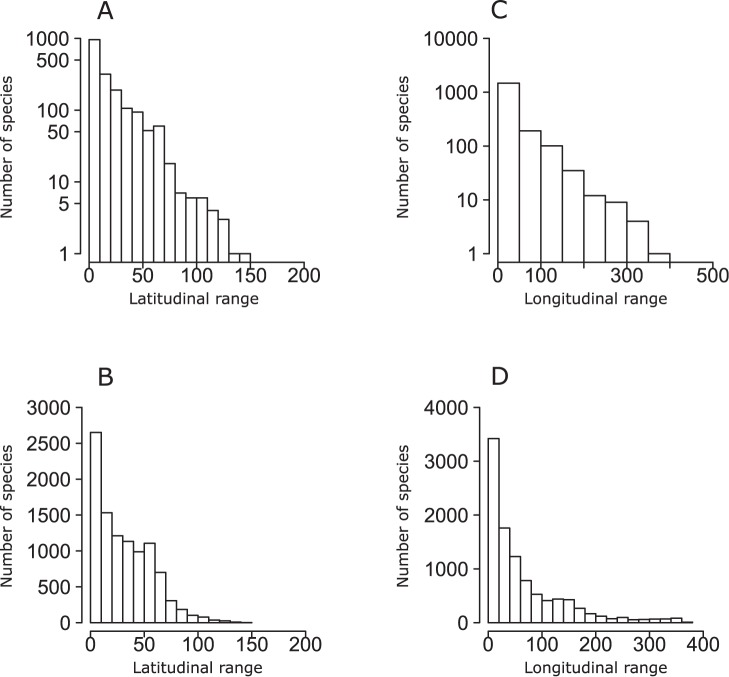
Frequency distribution plots of both maximum latitudinal (A and B) and longitudinal (C and D) ranges for freshwater (A and C) and marine (B and D) fishes.

**Figure 4:**
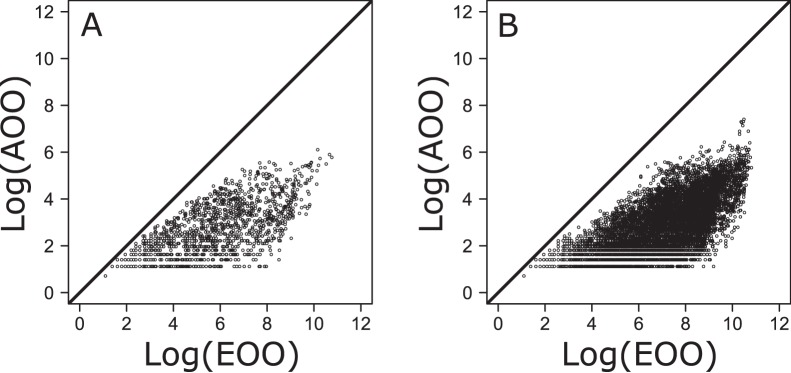
Relationship between fish species extent of occurrence (EOO) and area of occupancy (AOO). Both variables are logarithmically transformed (A: freshwater dataset; B: marine dataset).

In the present paper, OBIS data were used to compile two geo-referenced lists of freshwater and marine fish species, containing 1829 and 10068 species, respectively. Each species was assigned to a realm (freshwater or marine) according to Fishbase [12]. Freshwater and marine datasets were analysed both simultaneously and separately. To evaluate the possible influence of species introductions (human assisted translocations), we performed the overall analyses also using a reduced dataset excluding introduced species. Because we obtained results very similar to those achieved including species subjected to introduction, we used the full data sets for all analyses.

**Table 1:** Results of canonical correlation analyses (CANCORs) for the full data set and for freshwater and marine fish species separately.

	All species		Freshwater fish		Marine fish	
	D 1	D 2	D 1	D 2	D 1	D 2
**R_c_**	0.576	0.133	0.528	0.128	0.571	0.131
**Root**	0.332	0.018	0.279	0.016	0.326	0.017
***χ*** **^2^**	4769.424	202.925	625.759	29.991	4145.858	175.027
**Df**	12	5	12	5	12	5
**P**	0	0	0	0	0	0
**Redundancy**	0.212	0.006	0.215	0.003	0.207	0.006

The total data set included 11344 species, of which 1829 were freshwater species and 10068 marine species (553 species were considered both marine and freshwater). An analysis conducted excluding 461 species subjected to introductions gave virtually identical results. CANCORs were conducted accounting for phylogenetic non independence.

D1: Dimension 1, D2: Dimension 2, R_c_: Canonical correlation coefficient; df: degrees of freedom.

**Table 2:** Canonical loadings for the first two dimensions of CANCORs for freshwater and marine fish species.

	All species		Freshwater fish		Marine fish	
	D1	D2	D1	D2	D1	D2
**AOO**	0.958	0.289	0.992	0.051	0.957	0.272
**EOO**	0.602	0.799	0.746	0.608	0.594	0.793
**K**	−0.454	−0.665	−0.454	−0.747	−0.465	−0.548
**L**	0.708	0.900	0.746	0.845	0.735	0.849
**T**	0.123	0.06	0.156	−0.094	0.128	0.066
**W**	0.826	−0.118	0.685	−0.312	0.817	−0.093
**Y**	0.474	0.589	0.484	0.623	0.490	0.586
**Ym**	0.43	0.513	0.427	0.632	0.446	0.477

CANCORs were conducted accounting for phylogenetic non indepence. AOO: area of occupancy (number of 1×1° grid cells from which a species was recorded); EOO: extent of occurrence (latitudinal range longitudinal range); K: growth rate; L: maximum length; T: trophic level; Ym: age at first maturity; Y: life span; W: frequency of occurrence. D1: Dimension 1, D2: Dimension 2.

Range size for each species was estimated using measures of area of occupancy (AOO) and extent of occurrence (EOO) [20]. AOOs were calculated as follows: for each species, we plotted all available point records on a global grid of 1×1° Latitude/longitude and then we counted the number of grid cells where the species was present. For the EOO we alternatively tested two different measures. In a first set of analyses, EOOs were expressed as the number of 1×1° grid cells given by the product of species latitudinal and longitudinal ranges. Latitudinal range (Lat) was calculated as the difference between maximum and minimum latitude of species occurrence. Longitudinal range (Lon) was calculated as the difference between maximum and minimum longitude of species occurrence. In a second set of analyses, we used Lat and Lon as two separate measures of EOO instead of combining them into a single measure of species range size. This helped us to disentangle the possible effect of climate on latitudinal species distribution [21] from the information provided by longitudinal ranges, which are likely to be less influenced by climatic gradients.

**Figure 5:**
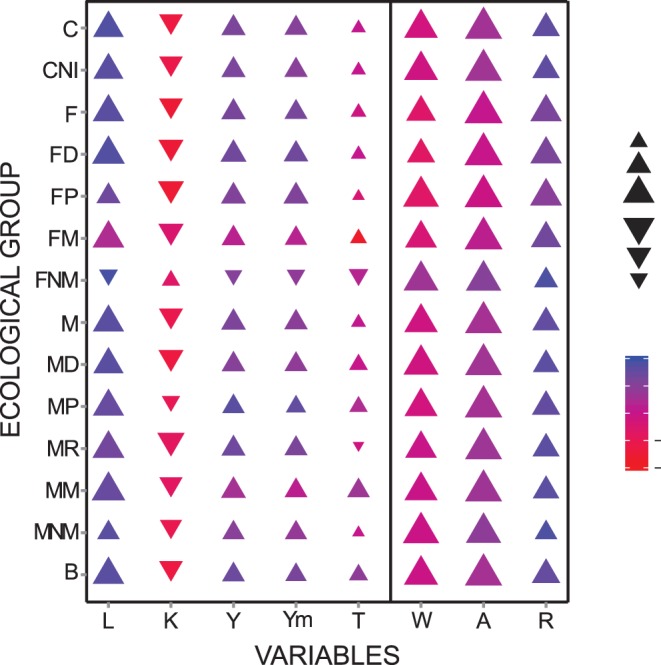
Relationships between canonical loadings of the first and second canonical dimensions for the sets of dependent and independent variables for all species, freshwater species, marine species and various ecological groupings. **Correlations with the first dimension are expressed by triangles (positive values) and reverse triangles (negative values). Correlations with the second dimension are expressed by colour scale.** AOO: area of occupancy (number of 1×1° grid cells from which a species was recorded); EOO: extent of occurrence (EOO, number of grid cells of 1° along the maximum latitudinal range × number of grid cells of 1° along the maximum longitudinal range of each species). Fish characteristics: L: maximum length; K: growth rate; T: trophic level; Ym: age at first maturity; Y: life span. Ecological categories: C: complete data set; CNI; complete data set excluding species subjected to be introduced; F: all freshwater species; FD: freshwater demersal; FP: freshwater pelagic; FM: freshwater migratory; FNM: freshwater non migratory; M: all marine species; MD: marine demersal; MP: marine pelagic; MM: marine migratory; MNM: marine non migratory; B: brackish.

To express dispersal and colonization ability we considered the following species traits of the adult phase of fishes: Maximum length (L: larger fishes are expected to have high dispersal power and to be less sensitive to predation than smaller fishes), growth rate coefficient of von Bertalanffy growth function (K: a low K value indicates that the species lives for many years and reaches slowly its maximum body size, whereas high K values are typical of short lived species with rapid growth; rapid growth is also characteristic of invasive species), age at first maturity (Ym: species which need more time to reach their sexual maturity are expected to travel more time before reproducing), life span (Y: the distance a fish can travel in a lifetime should increase with life span) and trophic level (T: species with higher trophic level are expected to have larger range for predation). L, K, Ym, Y and T were extracted for each species from Fishbase Species Ecology Matrices using a script based on the Python HTML/XML parser Beautiful Soup (http://www.crummy.com/software/BeautifulSoup). Specific details about these ecological parameters and the rationale for their use as measures of dispersal and colonization ability can be found in [22] and in Fishbase online documentation at http://www.fishbase.org/manual/Key%20Facts.htm. We also calculated for each species a measure of frequency of occurrence (W) as the ratio between its total number of point records and the number of grid cells where it occurs. This measure might reflect an estimate of overall abundance. However, because of unequal sampling among areas, this measure might also express differences in study efforts.

**Figure 6:**
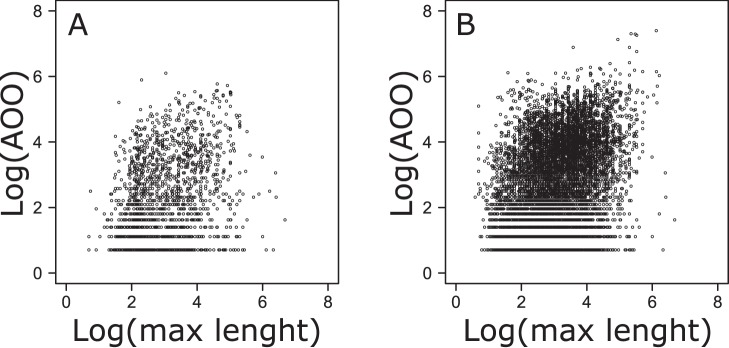
Relationship between fish species maximum body length and area of occupancy (AOO). Both variables are logarithmically transformed (A: freshwater dataset; B: marine dataset).

We performed a Canonical Correlation Analysis (CANCOR), using the aforementioned ecological parameters and the estimate of frequency of occurrence as a set of dependent variables, and range size measures (AOA and EOO) as a set of independent variables. CANCOR has several advantages over other multivariate techniques. Most notably, it enables the researcher to combine into a composite measure what otherwise might be an unmanageably large number of bivariate correlations, thus limiting the probability of committing Type I errors by using the same variables for too many statistical tests [23]. CANCOR is also particularly useful when the data researcher has little a priori knowledge about relationships among the sets of variables. Moreover, CANCOR is unique in developing multiple canonical functions independent one from another and thus able to represent different relationships among the sets of dependent and independent variables [24]. Normal q-q plots showed deviations from normality for all dependent and independent variables [25] which were therefore log-transformed to achieve normality [26]. Multivariate normal distribution in both dependent and independent variables was assessed using R function ‘mvnorm.etest’ from the package ‘energy’ [27], which implements a test based on Euclidean distance between sample elements [28].

Phylogentic relationships may generate pseudo-replication, because taxa that are similar by common descent inflate the sample size if they are counted as completely independent data points. The most used method to account for phylogeny is that of the independent contrasts [29]. To introduce the phylogenetic information into CANCORs with independent contrasts, we used the method called PCCA (Phylogenetic Canonical Correlation Analysis), with λ set at 1, which corresponds to a CANCOR performed on the phylogenetically independent contrasts [30]. To express phylogenetic relationships between species, we converted taxonomic arrangements (species, genus, subfamily, family, order) into a tree using the R library APE [31]. Branch lengths were assigned using Grafen’s method based on number of descendant observed taxonomic units, followed by Grafen’s ρ transform using ρ = 1 [32]. For comparative purposes, we also performed standard CANCORS (i.e. without accounting for phylogeny). All CANCORs (both including and excluding phylogeny) were performed using the R package ‘phytools’ [33]. Because we obtained similar results, we report here only those achieved accounting for phylogeny. Amount of variance in the set of dependent variables explained by that of independent variables was evaluated using redundancy analysis. Effects of dependent and independent variables on the respective canonical variates were evaluated by examining canonical loadings. To obtain robust CANCOR results the observations:variables ratios should be of at least 10∶1 [34], [35]. This requirement was fully satisfied in our study, with a ratio larger than 1000∶1 for the marine dataset, and larger than 100∶1 for the freshwater dataset.

In addition to the main subdivision between freshwater and marine species, we used a more detailed ecological categorization and performed separate CANCORs for each of the following ten categories using data from Fishbase [12]:

1. Marine migratory (including oceanodromous, anadromous, catadromous, potamodromous, amphidromous species);
2. Marine non migratory;
3. Marine demersal (including demersal, benthopelagic, and bathydemersal);
4. Marine reef-associated;
5. Marine pelagic (including bathypelagic, pelagic-neritic and pelagic-oceanic);
6. Freshwater migratory (including amphidromous, potamodromous, anadromous, catadromous, oceanodromous);
7. Freshwater non migratory;
8. Freshwater demersal (including demersal and benthopelagic);
9. Freshwater pelagic (including pelagic-neritic and pelagic).
10. Brackish

## Results

Frequency distribution plots of geographic range sizes expressed as area of occupancy (AOO) revealed that most geographic ranges tend to be relatively small in both marine and freshwater fish species (Fig. 1). Similar patterns were observed using extent of occurrences (EOO) (Fig. 2–3). Moreover, the use of the product of longitudinal and latitudinal ranges of species distribution (Fig. 2) or its decomposition in longitudinal and latitudinal ranges (Fig. 3) produced similar right skewed distributions for both marine and freshwater species. Plots of AOO against EOO for both marine and freshwater fishes (Fig. 4) showed that most species tend to have an AOO much smaller than the respective EOO.

Canonical Correlation Analysis (CANCOR) revealed strong correlations between range size and various fish traits, including maximum length, growth rate, age at first maturity, life span and trophic level. CANCOR results obtained using a combined measure of EOO (given by latitude range ×longitude range) and those obtained by decomposing it in two separate measures of latitudinal and longitudinal range were almost identical. Therefore we report only the results for the combined EOO measure (Tables 1 and 2).

In both marine and freshwater species, canonical correlation coefficients for the two dimensions were significant (P*<*0.0001). However, because a large number of observations could produce artificially low probability values for the canonical correlation coefficients, it was important to focus on the canonical roots. For both datasets, canonical roots indicated that the first dimension was particularly important. This was confirmed by the corresponding redundancy indexes, which indicated that the independent variables explained a substantial portion of variance of the dependent variables.

Canonical loadings of fish traits for the first and the second dimensions showed strict correlations between freshwater and marine datasets (Spearman coefficients r = 0.943, P*<*0.005 in both cases), thus indicating similar responses in marine and freshwater species. Canonical loadings of the first canonical dimension indicated that frequency of occurrence is the most influential parameter affecting fish range size in the overall data set and in the marine species, also being important for the freshwater species. Body length was the most important parameter in the freshwater data set, and the second most important parameter in the total and marine datasets. The importance of this variable was also shown by the second dimension. Although results associated with the second dimension should be considered with caution, it is important to note that area of occupancy was mainly associated with the first dimension, whereas, the extent of occurrence was mainly associated with the second dimension in the overall and marine datasets. The signs of the canonical loadings suggest that the influence of the parameter K (growth rate) on species distribution is opposite to those of all the other parameters (see also Fig. 5). Therefore, CANCOR results indicate that for both freshwater and marine fish species, range size is positively related to frequency of occurrence, body size, life span, age at first maturity and trophic level, whereas low growth rate is associated with narrower range size.

When species were subdivided into narrower ecological groups, CANCORs produced substantially similar results (Fig. 5, Information S1 and S2). For freshwater species, area of occupancy was mainly correlated with the first dimension in all ecological categories. Similarly, extent of occurrence was mainly correlated with the first dimension in all categories except for the non migratory species, where there was a strong correlation with the second dimension. The most important parameters highlighted by the first dimension were maximum length (especially in demersal and migratory species) and frequency of occurrence.

In the marine categories, area of occupancy was always strictly correlated with the first dimension, whereas, the extent of occurrence was correlated with the second dimension. The most important parameters highlighted by the first dimension were maximum length (especially in migratory species) and frequency of occurrence (especially in non migratory species).

## Discussion

One of the most documented macroecological patterns, exhibited by terrestrial, freshwater and marine organisms in a number of different ecosystems and geographical regions, is the distinctively unimodal, right skewed frequency distribution of the sizes of species geographical ranges [36], [37]. Our results for freshwater and marine fish species corroborate the idea that this pattern is really ubiquitous also at the global scale, showing that most fish species tend to have relatively small areas of occupancy, latitudinal ranges, longitudinal ranges and extents of occurrence. We obtained consistently similar frequency distribution patterns independently from the particular measure of distribution size used.

A fundamental relationship between area of occupancy and extent of occurrence of a species is that area of occupancy, being the space within the distributional limits of the species where its populations actually occur, should always be smaller than extent of occurrence [20]. Considering how we measured AOOs and EOOs, the consistency of our results with this prediction is not surprising. Yet, it is interesting to notice that in both marine and freshwater fish, most species tend to have an area of occupancy much smaller than their extent of occurrence (see Fig. 4), showing that only a relatively small fraction of the area that lies within the marginal limits of their distribution is really suitable and can be successfully occupied.

In general, CANCOR results indicate that for both freshwater and marine fish species, small-bodied species with high growth rate, small populations, short life span, reduced age at first maturity and low trophic level have a narrow range size. A positive correlation between species local abundance and range size is well documented in many taxa [38]. As our measure of frequency of occurrence may reflect abundance, its influence in marine and freshwater fish is not surprising, although we cannot definitively exclude that our measure of our measure of frequency of occurrence may also reflect study effort.

Besides the effect of frequency of occurrence, fish size emerged as a major determinant of range size, especially for marine migratory species. A positive correlation between body size and range size has been previously observed at large scale for several animal taxa [39], including a fish genus [40]. However, contrary to previous findings [36], [39], the spatial relationship between log(range size) and log(body size) found in marine and freshwater fish is not triangular, since not only small sized, but also many medium to large sized species exhibit great variability in their AOO values (see Fig. 6).

The overall positive correlations of range size with our measure of frequency of occurrence and body size support previous observations for the population size of North American Centrarchidae and Catostomidae [41], broadening the relevance of these patterns to a global scale and, more remarkably, extending them to the marine realm.

Recent studies suggest that home-range size and dispersal ability of terrestrial species tend to be associated because they are both measures of species mobility [42], [43]. Although some circumstantial evidence has been reported [44], this association has not been previously demonstrated for fish. Because body size is usually a good predictor of home-range [45], [46], our results provide some indirect support to this association. In our analyses, range size was defined using a measure of AOO and two distinct measures of EOO. Despite the common assumption of superior accuracy of AOO over EOO, both AOOs and EOOs are informative [20]. In both freshwater and marine fishes, however, EOO resulted to be less correlated with the independent variables than AOO.

The striking comparability of the results obtained for freshwater and marine fishes suggests that the main biogeographical patterns of fish in the marine and freshwater realms could be more similar than commonly assumed. The study of which ecological traits of fish species may influence their distribution is generally hampered by a number of factors, including species introductions and adult migrations. Marine fish introductions are common and can lead to severe ecological consequences [38], but in general are difficult to track. For example, unrecognized introductions can lead the researcher to erroneously consider as cosmopolitan a species which, in fact, has been introduced [39]. To avoid the confounding effect of adult migrations, researches dealing with fish ranges have been mostly focused on reef fish species, because most of them have a sedentary adult life style, and suitable habitats are considered sufficiently isolated to make colonization processes only possible through larval migration [5]. However, the geographic distance between reefs may be small compared to larval drift distances, and only species that have limited larval drift are really isolated. Thus, internal recruitment [10] and the lack of habitat isolation in reefs [9] may introduce important biases. Freshwater species are even more problematic because of the role played by geographical constraints and hydrographic events in their range expansion [7] and by human introductions. However, these problems did not affect the aim of our study. We were interested in searching for those species traits which concur to determine colonization ability and hence regulate range size. In doing so, we were not concerned to establish if a given occurrence was due to a native or an introduced population, or if it was due to hydrographic events or adult migrations, because any species which reaches a new area would face similar difficulties to succeed in colonization independently from how it gets there. Thus, the relationships between range size and species traits found in our study cannot be associated with particular mechanisms of dispersal, but reflect the influence of species colonization ability.

One might object that human assisted translocation has been an overwhelmingly greater mechanism for dispersal in freshwater as opposed to coastal marine fish communities, and that a comparison of the colonization ability of these two groups of fishes is confounded by that phenomenon. However our analysis showed that omitting species known to be subject to introduction does not alter the results, thus we think that the patterns found in this study are “natural”.

The amount of analysed freshwater fishes for pelagic and non migratory categories was much smaller than the species used for marine categories, which might have biased some particular results of our study. Thus, it would be important, in the future, to test especially freshwater fish data for potential bias in data accuracy, for example using representative samples of continent specific and lentic vs. lotic fish communities. However, the very large total number of freshwater species considered in this study and the high concordance of results obtained for freshwater species with different ecologies, suggests that such biases do not have a strong influence on our general conclusions.

## Supporting Information

Information S1 **Results of canonical correlation analysis (CANCOR) for freshwater fish species divided into ecological categories.**
(PDF)

Information S2 **Results of canonical correlation analysis (CANCOR) for marine fish species divided into ecological categories.**
(PDF)
