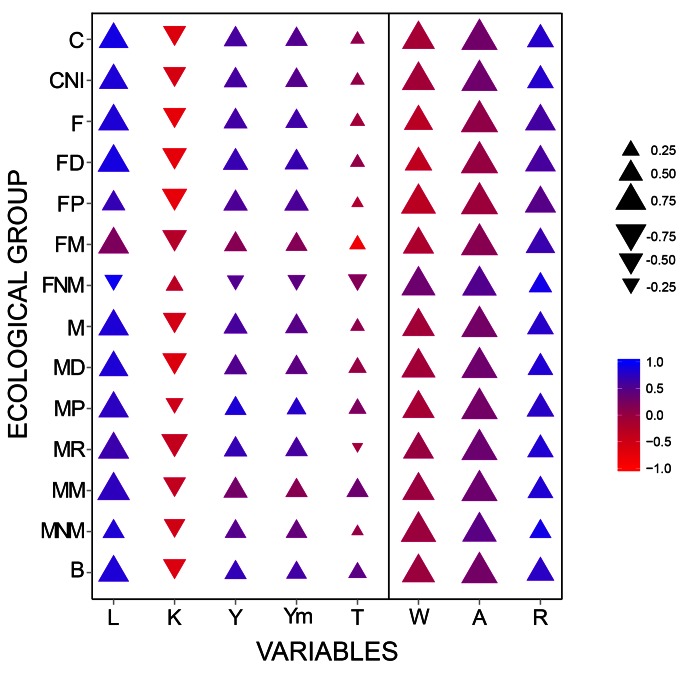# Correction: Global-Scale Relationships between Colonization Ability and Range Size in Marine and Freshwater Fish

**DOI:** 10.1371/annotation/30f3e1d0-5e35-4cf0-b062-56400a5f13a4

**Published:** 2013-05-22

**Authors:** Giovanni Strona, Paolo Galli, Simone Montano, Davide Seveso, Simone Fattorini

There was an error in Figure 5. The correct version of the figures is available here: 

**Figure:**