# Supplementary material for: Global-Scale Relationships between Colonization Ability and Range Size in Marine and Freshwater Fish
Source: PLoS One. 2012 Nov 21;7(11):e49465. doi: 10.1371/journal.pone.0049465 (PMC3504041; doi:10.1371/journal.pone.0049465)
Supplement: Information S1 — Results of canonical correlation analysis (CANCOR) for freshwater fish species divided into ecological categories. (PDF) [file pone.0049465.s001.pdf]

Information S1 Results of canonical correlation analysis (CANCOR) for freshwater fish species divided into ecological categories.

CANCORs were conducted accounting for phylogenetic non independence in all analyses.

	Demersal (1590 species)		Pelagic (186 species)		Migratory (598 species)		Non migratory (108 species)	
	D1	D2	D1	D2	D1	D2	D1	D2
Canonical correlation coefficient	0.549	0.136	0.603	0.188	0.462	0.159	0.620	0.360
Root	0.301	0.019	0.363	0.035	0.214	0.025	0.385	0.130
χ^2	598.075	29.733	88.05	6.512	157.53	15.137	64.048	14.233
df	12	5	12	5	12	5	12	5
P	0	0	0	0.260	0	0.010	0	0.014
Redundancy	0.232	0.003	0.283	0.004	0.156	0.006	0.194	0.071
Canonical loadings								
AOO	0.994	0.006	0.988	-0.048	0.983	0.123	0.891	0.509
EOO	0.741	0.588	0.762	0.482	0.700	0.670	0.465	0.912
K	-0.505	-0.739	-0.536	-0.77	-0.512	-0.256	0.248	-0.341
L	0.789	0.889	0.471	0.706	0.751	0.206	-0.270	0.943
T	0.142	0.044	0.042	-0.234	0.201	-0.844	-0.300	0.161
W	0.622	-0.359	0.904	-0.339	0.762	-0.198	0.870	0.318
Y	0.542	0.686	0.503	0.547	0.453	0.131	-0.229	0.511
Ym	0.485	0.684	0.508	0.555	0.411	0.151	-0.259	0.430

AOO: area of occupancy (number of 1×1° grid cells from which a species was recorded); EOO: extent of occurrence (latitudinal range longitudinal range); K: growth rate; L: maximum length; T: trophic level; Ym: age at first maturity; Y: life span; W: frequency of occurrence. D1: Dimension 1, D2: Dimension 2.
