# Supplementary material for: Global-Scale Relationships between Colonization Ability and Range Size in Marine and Freshwater Fish
Source: PLoS One. 2012 Nov 21;7(11):e49465. doi: 10.1371/journal.pone.0049465 (PMC3504041; doi:10.1371/journal.pone.0049465)
Supplement: Information S2 — Results of canonical correlation analysis (CANCOR) for marine fish species divided into ecological categories. (PDF) [file pone.0049465.s002.pdf]

Information S2 Results of canonical correlation analysis (CANCOR) for marine fish species divided into ecological categories.

CANCORs were conducted accounting for phylogenetic non independence in all analyses.

	Demersal (5176 species)		Pelagic (1517 species)		Reef (3373 species)		Migratory (1087 species)		Non migratory (879 species)		Brackish (2093 species)	
	D1	D2	D1	D2	D1	D2	D1	D2	D1	D2	D1	D2
Canonical correlation coefficient	0.597	0.153	0.589	0.137	0.541	0.105	0.568	0.112	0.591	0.181	0.571	0.154
Root	0.357	0.023	0.347	0.019	0.293	0.011	0.323	0.012	0.350	0.033	0.326	0.024
χ^2	2404.837	122.412	671.789	28.609	1203.214	37.634	435.375	13.556	404.764	29.079	873.426	50.165
df	12	5	12	5	12	5	12	5	12	5	12	5
P	0	0	0	0	0	0	0	0.019	0	0	0	0
Redundancy	0.214	0.009	0.229	0.006	0.182	0.004	0.197	0.005	0.168	0.017	0.216	0.008
Canonical loadings												
AOO	0.948	0.310	0.965	0.271	0.951	0.337	0.953	0.318	0.888	0.457	0.962	0.269
EOO	0.549	0.830	0.626	0.786	0.582	0.831	0.560	0.836	0.416	0.908	0.632	0.773
K	-0.506	-0.659	-0.252	-0.496	-0.587	-0.412	-0.437	-0.386	-0.392	-0.533	-0.452	-0.645
L	0.700	0.842	0.732	0.758	0.762	0.659	0.821	0.748	0.416	0.840	0.749	0.847
T	0.268	0.006	0.256	0.210	-0.001	-0.097	0.417	0.330	0.042	0.020	0.282	0.453
W	0.877	-0.096	0.806	-0.118	0.752	-0.013	0.808	-0.015	0.924	-0.038	0.839	-0.040
Y	0.455	0.509	0.410	0.851	0.469	0.701	0.497	0.276	0.402	0.497	0.423	0.718
Ym	0.43	0.433	0.327	0.794	0.434	0.592	0.422	0.120	0.398	0.390	0.367	0.617

AOO: area of occupancy (number of 1×1° grid cells from which a species was recorded); EOO: extent of occurrence (latitudinal range longitudinal range); K: growth rate; L: maximum length; T: trophic level; Ym: age at first maturity; Y: life span; W: frequency of occurrence. D1: Dimension 1, D2: Dimension 2.
